# *SHB1/HY1* Alleviates Excess Boron Stress by Increasing *BOR4* Expression Level and Maintaining Boron Homeostasis in Arabidopsis Roots

**DOI:** 10.3389/fpls.2017.00790

**Published:** 2017-05-16

**Authors:** Qiang Lv, Lei Wang, Jin-Zheng Wang, Peng Li, Yu-Li Chen, Jing Du, Yi-Kun He, Fang Bao

**Affiliations:** College of Life Sciences, Capital Normal UniversityBeijing, China

**Keywords:** *Arabidopsis thaliana*, heme oxygenase 1, carbon monoxide, bilirubin, BOR4, primary root elongation, excessive boron tolerance

## Abstract

Boron is an essential mineral nutrient for higher plant growth and development. However, excessive amounts of boron can be toxic. Here, we report on the characterization of an Arabidopsis mutant, *shb1* (*sensitive to high-level of boron 1*), which exhibits hypersensitivity to excessive boron in roots. Positional cloning demonstrated that the *shb1* mutant bears a point mutation in a gene encoding a heme oxygenase 1 (HO1) corresponding to the *HY1* gene involved in photomorphogenesis. The transcription level of the *SHB1/HY1* gene in roots is up-regulated under excessive boron stimulation. Either overexpressing *SHB1/HY1* or applying the HO1 inducer hematin reduces boron accumulation in roots and confers high boron tolerance. Furthermore, carbon monoxide and bilirubin, catalytic products of HO1, partially rescue the boron toxicity-induced inhibition of primary root growth in *shb1*. Additionally, the mRNA level of *BOR4*, a boron efflux transporter, is reduced in *shb1* roots with high levels of boron supplementation, and hematin cannot relieve the boron toxicity-induced root inhibition in *bor4* mutants. Taken together, our study reveals that HO1 acts via its catalytic by-products to promote tolerance of excessive boron by up-regulating the transcription of the *BOR4* gene and therefore promoting the exclusion of excessive boron in root cells.

## Introduction

Boron (B), with low abundance but wide distribution in nature, is an essential micronutrient for plants and animals (Warington, 1923; Nielsen, 2008). Both boric acid and borate are able to form complexes involved in different cellular processes (Cakmak et al., 1995; Findeklee and Goldbach, 1996). Until now, the most widely accepted function of B is the formation of pectic substances. Borate crosslinks with apiose residues of rhamnogalacturonan II (RG-II), and this complex plays an essential role in cell wall structure and function (Kobayashi et al., 1996; O’Neill et al., 2004). Other functions of B have also been suggested, such as maintenance of membrane integrity, and metabolism of biomacromolecules (nucleic acids, carbohydrates, and proteins, etc.) (Blevins and Lukaszewski, 1998; Reid et al., 2004).

Boron is taken up from soil by roots in the form of boric acid and is essential for plant growth within a narrow optimal range. Deficiency of boron results in severe defects in vegetative and reproductive growth (Shorrocks, 1997), while excessive boron causes toxic effects (Nable et al., 1997). Hence, it is important for plants to maintain a boron homeostasis for normal growth and development, and a complex system might be employed to regulate the absorption, mobility, distribution, and storage of B (Hänsch and Mendel, 2009).

Boron toxicity is a serious agricultural problem in semi-arid areas all over the world (Camacho-Cristóbal et al., 2008). Toxic effects of boron might include inhibition of cell division and elongation, defects in cell wall development, and metabolic disruption (Reid et al., 2004). Although the toxicity symptoms can be diverse in aerial parts of plants, high boron has been reported to inhibit root growth of many species, including barley, Arabidopsis, and maize, by reducing the size of root meristems (Choi et al., 2007; Aquea et al., 2012; Esim et al., 2013).

Many plant species can reduce the toxic effects of excessive boron by reducing boron accumulation via root boron exclusion (Hayes and Reid, 2004; Hamurcu et al., 2016). Several transporter-encoding genes have been proven to play roles in tolerance to excessive boron, such as *BOR4* in Arabidopsis (Miwa et al., 2007), *Bot1* in barley (Sutton et al., 2007), and *Bo1* and *Bo4* in wheat (Pallotta et al., 2014). To date, the mechanism of boron uptake and distribution in plants has been well studied; however, our knowledge of the regulation mechanism for maintaining B homeostasis is still limited.

Heme oxygenase (HO; EC 1.14.99.3) is the rate-determining enzyme in the transformation of heme into biliverdin, which is then converted into bilirubin (BR) by biliverdin reductase. Heme degradation is accompanied by the release of free Fe and carbonic oxide (CO) (Wilks, 2002). Previous reports indicate that homologs of HO exist widely in different plant species, including Arabidopsis, rice, pea, tobacco, soybean, and tomato, similar to those found in humans, suggesting the evolutionary conservation of *HO* genes (Shekhawat and Verma, 2010).

Muramoto et al. (1999) cloned the first Arabidopsis HO-encoding gene, *HY1*. Subsequently, three other HOs (HO2, HO3, and HO4) were identified in the Arabidopsis genome. These four members of the HO family fall into two subfamilies: one subfamily contains *HY1/HO1, HO3*, and *HO4*, while *HO2* belongs to another subfamily (Gisk et al., 2010). Among them, *HO1* has much higher mRNA abundance than the others and can be induced by various chemical and stress stimuli, such as salinity (Xie et al., 2011, 2013), heavy metals (Han et al., 2008, 2014), UV-B and UV-C (Yannarelli et al., 2006; Xie et al., 2012), H_2_O_2_ (Chen et al., 2009), and iron deficiency (Li et al., 2013; Yang et al., 2016). These data imply the indispensable protection roles of HOs and their enzymatic products in plant growth and development processes under hostile environmental conditions.

In this study, we identified an Arabidopsis mutant named s*hb1* (*sensitive to high-level of boron 1*) that showed major developmental defects in roots under high concentrations of boron. Positional cloning of the *SHB1* gene indicated that the causative locus was an allele of the *Arabidopsis thaliana HY1* gene. Pharmacological, genetic, and molecular evidence showed that SHB1/HY1 plays an essential role in the tolerance of B toxicity.

## Materials and Methods

### Plant Materials and Growth Conditions

All of the Arabidopsis mutagenic or T-DNA insertion mutants and transgenic plants used in this study were in a Col-0 (Columbia) background except for *phyAphyB*, which was in a Ler (Landsberg) background. The mutants *hy1-100* (CS236), *phyAphyB* (CS6224), *ho2* (SALK_113008), *ho3* (SALK_034321), *ho4* (SALK_044934), *bor4-1* (SALK_135095), and *bor4-5* (WiscDsLox233D10) were obtained from the ABRC (Arabidopsis Biological Resource Center). The *hy1-101* (Zhai et al., 2007) and *hy5-215* (Oyama et al., 1997) mutants were described previously.

The seeds were surface sterilized with 10% sodium hypochlorite solution for 10 min and washed five times with sterile water, then grown on solid half-strength Murashige and Skoog (1/2 MS) medium (Sigma-Aldrich) supplemented with 1% (w/v) sucrose, 0.05% (w/v) MES, and 1% (w/v) agar, with or without boric acid. After cold treatment at 4°C for 2–4 days in the dark, the plates were transferred into a controlled growth chamber at 21–22°C under cool-white fluorescent light (80–100 μmol m^-2^ s^-1^) in a long-day photoperiod (16 h light/8 h dark).

### Mutant Screening

Ethyl methanesulfonate-mutagenized M2 Col-0 seeds were grown on 1/2 MS medium supplemented with 2 mM B and seedlings were screened for inhibited primary root growth. The *shb1* mutant after three rounds of backcrossing to Col-0 wild-type was used for phenotype analysis and the generation of a mapping population.

### Map-Based Cloning

For map-based cloning of the *shb1* locus, 716 F2 mapping plants segregated from the cross of the *shb1* mutant and wild-type Ler were used to delimit the boundaries of the locus with simple sequence length polymorphism markers as listed in Supplementary Table S1 (Pǎcurar et al., 2012).

### Seed Germination Measurement

Germination assay was performed as described by Millar et al. (2006). Seeds germinating on plates containing various boron concentrations were scored, and germination proportion was analyzed at different time points.

### RNA Isolation and Gene Expression Analysis

mRNA levels were measured by quantitative RT-PCR and semi-quantitative RT-PCR. Total RNA was extracted from the plant materials using Trizol Reagent (Invitrogen) according to the manufacturer’s instructions. Light absorption values of RNA were measured using a NanoDrop spectrophotometer. The overall quality of total RNA was evaluated by formaldehyde–RNA denaturing electrophoresis and the A260/A280 ratio.

First-strand cDNA was synthesized with 1 μg of total RNA using M-MLV reverse transcriptase and an oligo(dT)15 primer (Takara) in a 10 μl mixture. The reaction was then diluted twofold with nuclease-free water, and the cDNA was used as a template for both semi-quantitative RT-PCR and quantitative RT-PCR. For the semi-quantitative PCR analysis, 1 μl of each reverse transcription reaction was used per PCR reaction in a final volume of 20 μl. Semi-quantitative RT-PCR was performed with gene-specific primers (Supplementary Table S1), and various cycle numbers were tested to determine the logarithmic phase of amplification for each gene. *AtACTIN8* was used as the reference gene. The RT-PCR experiments were repeated three times independently, and similar results were obtained. Real-time PCR was performed using the SYBR green PCR master mix (Takara) and run in a Bio-Rad iQ5 real-time PCR detection system. *AtACTIN8* was used as the reference (Ohkama-Ohtsu et al., 2004). The primer sequences were given in Supplementary Table S1. All reactions were repeated at least three times. Statistical analysis of the results of real-time PCR was performed using the 2^-ΔΔCt^ method.

### Plasmid Constructions and Plant Transformation

*pCAMBIA1302* was used to generate *p35S::SHB1:GFP* (green fluorescent protein) and *pSHB1::SHB1:GFP* constructs. A 846 bp fragment of the *SHB1* coding region was obtained by amplifying from the wild-type cDNA using the following primers: *SHB1*-*NcoI*-F (5′- CATGCCATGGCGTATTTAGCTCCGA-3′) and *SHB1*-*SpeI*-R (5′-GACTAGTGGACAATATGAGACGAAGTATCTC-3′). Restriction sites for *Nco*I and *Spe*I are underlined. Primers *pSHB1*-*HindIII*-F (5′-CCCAAGCTTTCTTCTCGTTGCCACCGTT-3′) and *pSHB1*-*NcoI*-R (5′-CATGCCATGGGGTTTGATCGGAATAGAAAAATG-3′) were used to amplify a genomic DNA fragment containing the *SHB1* promoter. The fragment (∼1200 bp) was amplified from wild-type genomic DNA, including *Hind*III and *Nco*I restriction sites (underlined). The promoter fragment was then recombined into *pCAMBIA1302* to obtain *pSHB1::SHB1:GFP*. Transgenic lines were generated using *Agrobacterium tumefaciens* LBA3101 by the floral dip method (Clough and Bent, 1998). Seeds of the T1 generation were sown on 1/2 MS medium containing 25 mg/L hygromycin B (Roche), and T3 homozygous lines were used for further experiments.

### Determination of B Content

Whole roots of 21-day-old seedlings were harvested from 100 to 200 individual plants grown vertically on medium containing 0.05 mM B and from 300 to 500 plants grown on medium containing 2 mM B. The samples were dried at 60°C for at least 3 days, digested with concentrated (13 M) nitric acid, and B content measured using inductively coupled plasma–mass spectrometry as previously described (Takano et al., 2002).

## Results

### The *shb1* Mutant is Hypersensitive to Excessive B Stress

**FIGURE 1:**
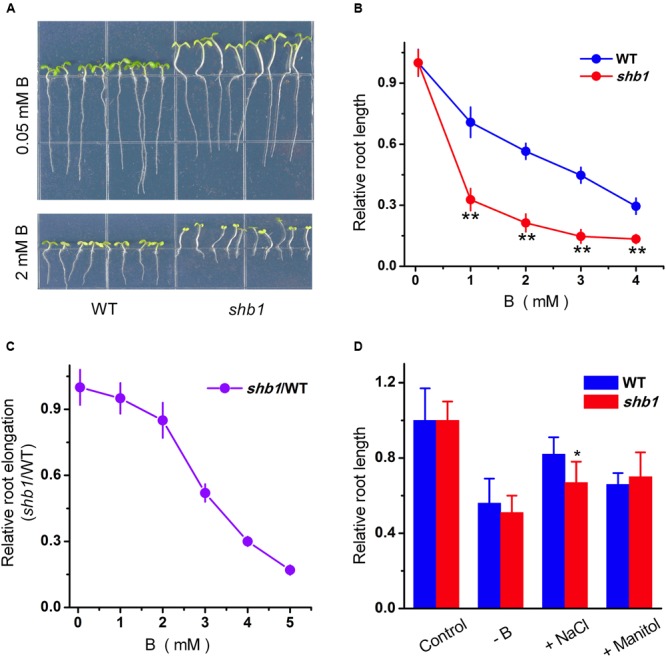
**Phenotypes of Arabidopsis *shb1* mutants under excessive boron stress. (A)** Wild-type Col-0 and *shb1* seedlings grown on medium containing 0.05 mM (top) or 2 mM (bottom) B for 10 days. **(B)** Dose-dependent effects of B on growth inhibition of roots in wild-type and *shb1* seedlings. The relative root length of 10-day-old seedlings grown on medium containing various concentrations of B (0.05, 1, 2, 3, or 4 mM) were measured. **(C)** Comparative analysis of *shb1* root elongation in response to B. Five-day-old wild-type and *shb1* seedlings were transferred to medium containing 0.05, 1, 2, 3, 4, and 5 mM B and incubated for another 7 days. The ratio between *shb1* and wild-type root elongation was plotted, and the ratio at 0.05 mM B was defined as 1. **(D)** Sensitivity of wild-type and *shb1* seedlings to other stress conditions such as B deficiency (0.003 mM B), osmotic stress (300 mM mannitol), and high salinity (90 mM NaCl). The relative root length data are expressed as means ± SD (*n* > 10) relative to the values obtained under normal conditions with three replicates for each concentration point. Asterisks indicate the significant difference between *shb1* mutants and wild-type under B treatments (Student’s *t*-test, ^∗^*P* < 0.05, ^∗∗^*P* < 0.01).

In order to identify novel boron tolerance-related mutants, we set up a genetic screen for Arabidopsis mutants with altered responses to high concentration of boric acid (2 mM) by root growth inhibition assay. A mutant named *shb1* (*sensitive to high-level of boron 1*) was initially isolated for its dramatically inhibited primary root growth under excessive boron treatment (**Figure 1A**).

Further characterization indicated that, on average, roots of the *shb1* seedlings were about 20% shorter than those of the wild-type seedlings when growing on regular MS medium (0.05 mM B) (**Figure 1A**) but were inhibited much more significantly in the presence of additional B from 1 to 4 mM (**Figures 1A,B**). Because excess B delayed wild-type seed germination, and decreased the germination ratio of *shb1* seeds by about 20% at the 72 h after planting (Supplementary Figures S1A,B), seedlings with similar growth status were transferred to MS plates containing different concentrations of boric acid to eliminate the interference of different germination rates, and the result was consistent with our previous observation that the primary root elongation of *shb1* was dramatically inhibited by high levels of B (**Figure 1C**).

In contrast to excessive boron treatments, the *shb1* seedlings only exhibited slightly higher primary root inhibition by high salinity than the wild-type seedlings, and no significant difference was observed between the mutant and wild-type roots under B deficiency and mannitol-induced osmotic stress (**Figure 1D**).

Long hypocotyl is another obvious phenotype of *shb1* (**Figure 1A**). However, the *shb1* hypocotyl growth was not affected by high concentrations of boron (**Figure 1A**). Thus this phenotype might not be related to B response.

### *SHB1* Encodes Heme Oxygenase 1, a Key Enzyme that Functions in Heme Metabolism

**FIGURE 2:**
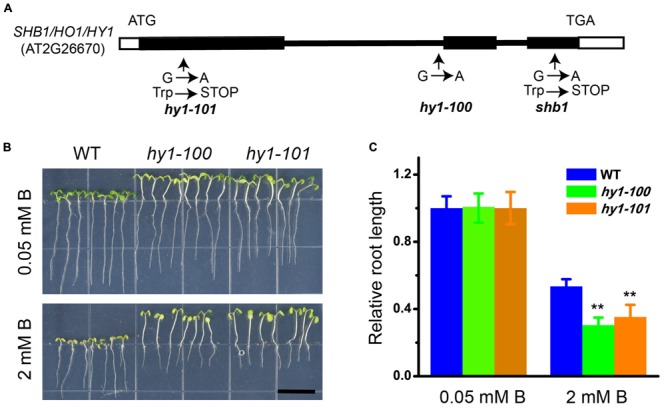
**Molecular characterization of *SHB1*. (A)** Diagram of *SHB1* genomic structure. Black and white boxes indicate coding regions and untranslated regions, respectively. Sites of the *shb1, hy1-100*, and *hy1-101* mutations are shown. **(B)** Excessive B-dependent short root phenotypes of *hy1-100* and *hy1-101* mutant seedlings are shown. Wild-type and mutant seedlings were grown on medium containing 0.05 mM (top) or 2 mM (bottom) B for 10 days. Bars = 1 cm. **(C)** Relative root length data as described in **(B)**. The relative root length data are expressed as means ± SD (*n* > 10) relative to the values obtained under normal conditions with three replicates for each concentration point. Asterisks indicate the significant difference between mutants *hy1-100, hy1-101*, and wild-type under B treatments (Student’s *t*-test, ^∗∗^*P* < 0.01).

Genetic analysis indicated that *shb1* was a single-gene recessive mutant, because the F1 plants from a cross between *shb1* and wild-type plants were normal, and F2 progeny segregated as wild-type to *shb1* mutants with a 3 to 1 ratio (Supplementary Figures S2A–C). *SHB1* was first mapped on chromosome 2 between the markers UPSC_2-1181 and UPSC_2-18980. Fine mapping delimited *SHB1* between markers UPSC_2-10851 and UPSC_2-11823 (Supplementary Figure S2D). One of the open reading frames within this interval is the previously reported *HY1* (AT2G26670), which encodes heme oxygenase 1 (HO1). Since the *hy1* mutant is morphologically similar to *shb1* (Davis et al., 1999; Muramoto et al., 1999; Zhai et al., 2007), *HY1* is a likely candidate for *SHB1*. Sequence analysis of genomic DNA fragments covering the *HY1* coding region in *shb1* revealed a single G to A nucleotide substitution resulting in the change of the tryptophan codon (TGG) to a stop codon (TGA) at amino acid 232, causing premature termination of the translated protein (**Figure 2A**).

Phenotype analysis of *hy1* alleles strongly supported our mapping and sequencing results. Both *hy1-100* and *hy1-101* (Davis et al., 1999; Zhai et al., 2007) exhibited similar phenotypes to that of *shb1*, with long hypocotyl and hypersensitivity of root growth to excessive B (**Figures 2B,C**).

Furthermore, a cDNA fragment containing the entire *HY1* coding region and a 1.2 kb upstream genomic DNA fragment was introduced into the *shb1* mutant for complementation testing. The transgenic plants showed no obvious difference from the wild-type plants in growth and development (Supplementary Figures S3A–C) on either normal or excessive B medium. These data indicate that the *shb1* mutant is a new allele of the *hy1* mutant.

There are four *HOs* in the Arabidopsis genome (Gisk et al., 2010), so we investigated the possible functional redundancy among these genes. Compared with the other three genes, *SHB1/HY1* mRNA is the most enriched in roots according to the public expression database and previous reports (Zimmermann et al., 2004; Xie et al., 2011). Furthermore, none of the *HO2, HO3*, or *HO4* T-DNA insertion mutants (SALK_113008, SALK_034321, and SALK_044934) showed a hypersensitivity to excessive boron (Supplementary Figure S4). So, HO1 might be the major HO involved in the response of roots to high boron stress.

### Promoting *SHB1/HY1* Expression Resulted in Better Tolerance to B Toxicity

**FIGURE 3:**
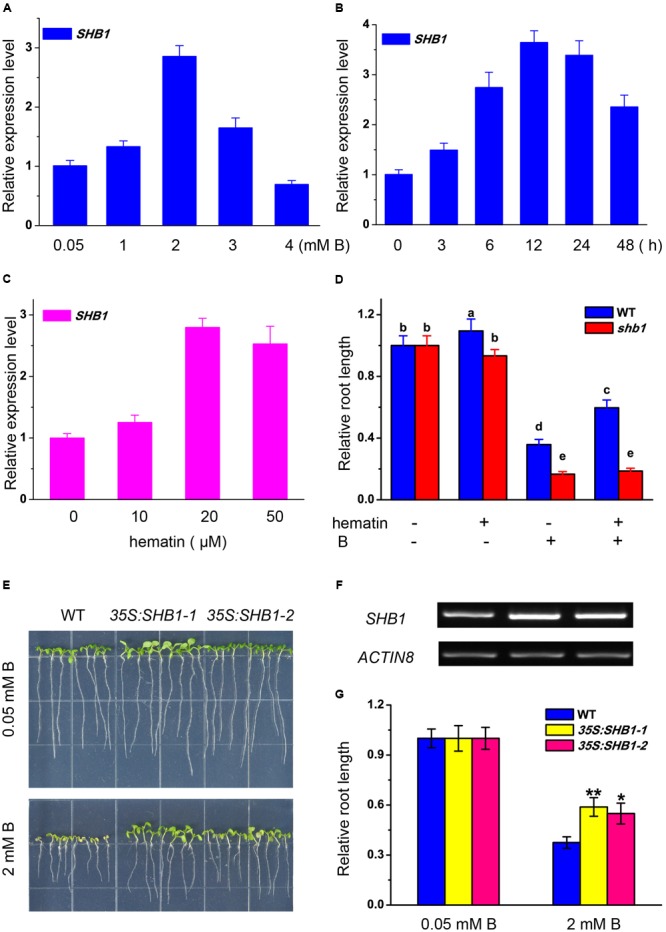
**Transcription analysis of *SHB1* in response to boron toxicity. (A,B)** Quantitative RT-PCR analysis of *SHB1* gene expression in wild-type seedlings in response to boron toxicity. **(A)** Five-day-old wild-type seedlings were transferred to medium containing 0.05, 1, 2, 3, and 4 mM B, respectively, and incubated for 24 h. Data were normalized to *ACTIN8* mRNA levels in the same samples and are expressed as means ± SD (*n* = 3). **(B)** Five-day-old wild-type seedlings were transferred to medium containing 2 mM B and incubated for 0, 3, 6, 12, 24, and 48 h, respectively. Data were normalized as described in **(A)**. **(C)** Quantitative RT-PCR analysis of *SHB1* gene expression in wild-type seedlings in response to hematin. Five-day-old wild-type seedlings were transferred to medium containing 0, 10, 20, and 50 μM hematin, respectively, and incubated for 3 days. Data were normalized as described in **(A)**. **(D)** Effects of excessive B on root elongation in wild-type and *shb1* seedlings with or without 20 μM hematin in the medium. Bars with different letters are significantly different at *P* < 0.05 according to Tukey’s multiple range test. **(E)** Morphology of wild-type and SHB1 over-expression lines (*35S:SHB1-1* and *35S:SHB1-2*) subjected to 2mM B for 10 days. **(F)** *SHB1* transcript levels in each *SHB1* over-expression line (*35S:SHB1-1* and *35S:SHB1-2*) under normal growth conditions for 10 days, analyzed by RT-PCR. *AtACTIN8* was used as the reference gene. **(G)** The relative primary root lengths of wild-type and *35S:SHB1-1* and *35S:SHB1-2* seedlings were measured after growth on medium containing 0.05 or 2 mM B for 10 days. All the data shown are expressed as means ± SD (*n* > 10) relative to the value obtained for wild-type plants grown under normal conditions (defined as 1), and asterisks represent significant differences (^∗^*P* < 0.05, ^∗∗^*P* < 0.01, Student’s *t*-test) relative to wild-type.label

The expression of *SHB1* in roots was regulated by boron stress in a dosage-dependent way. *SHB1/HY1* transcription was induced by up to nearly three times that of the control under mild boron stress (2 mM) (**Figure 3A**). The expression of *SHB1* was significantly induced by 2 mM B at 6 h, peaked at 12 h, and was still more than one fold higher than that of the control after 48 h of treatment (**Figure 3B**).

Hematin is a potential inducer of HO1 and was reported to promote *HY1* transcription (**Figure 3C**) (Xie et al., 2011). Application of hematin partially rescued the growth inhibition of the wild-type roots caused by excessive boron, but had no effect on *shb1* roots (**Figure 3D**).

Moreover, the primary roots of *SHB1/HY1* over-expression seedlings (*35S:SHB1-1* and *35S:SHB1-2*) were significantly longer than those of the wild-type when grown on medium containing 2 mM boron (**Figures 3E–G**), indicating that a high level of *SHB1/HY1* expression could alleviate the inhibition of primary root elongation induced by excessive boron.

### The Enzymatic By-Products of HO1, CO, and BR Confer Tolerance to B Stress

**FIGURE 4:**
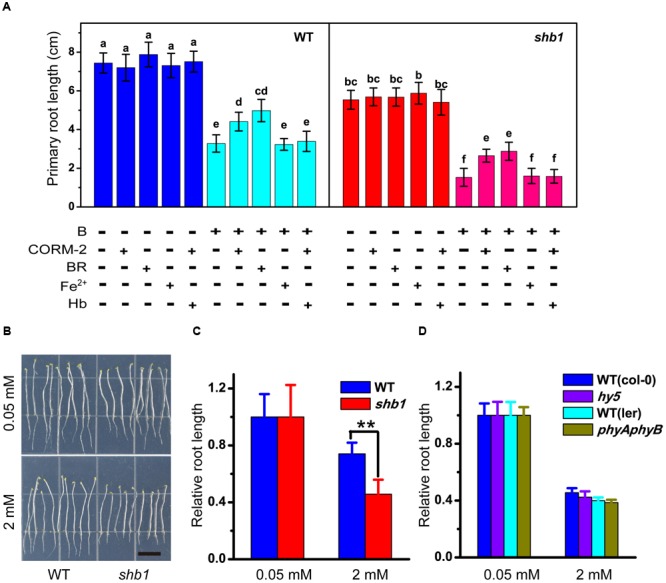
**Pharmacological assays of excessive B tolerance in roots. (A)** Primary root length of wild-type and *shb1* on medium containing varying concentrations of B, 50 μM CORM-2, 10 μM BR, 50 μM Fe-EDTA, 50 μM hemoglobin (Hb) or a combination of treatments for 10 days. **(B)** Sensitive phenotype of *shb1* in the skotomorphogenesis process. Wild-type and *shb1* seedlings growing on medium containg 2 mM B without light for 10 days. Bars = 1 cm. **(C)** Relative root length of wild-type and *shb1* seedlings grown as described in **(B)**. **(D)** Relative root length of light signal-related mutants *hy5* and *phyAphyB* on medium containing 0.05 and 2 mM B. All values are means ± SD of three different experiments with at least three replicated measurements. Bars with different letters are significantly different at *P* < 0.05 according to Tukey’s multiple range test in **(A)**, and asterisks represent significant differences (^∗∗^*P* < 0.01, Student’s *t*-test) relative to wild-type in **(C)**.

Because HO1 catalyzes the conversion of heme into biliverdin, along with the producing of carbon monoxide (CO) and free iron (Fe^2+^), we speculated that HO1 might function via its by-products. We therefore tested the effects of BR (the subsequent product of biliverdin IXa), CORM-2 (a CO donor), and Fe-EDTA on B tolerance of the root. Application of Fe-EDTA showed no significant effect on root growth (**Figure 4A**), while both CORM-2 and BR partially alleviated excessive B-induced inhibition of primary root elongation in wild-type and *shb1* seedlings (**Figure 4A**). On the other hand, hemoglobin (a CO scavenger) could eliminate the CORM-2 effect of restoring root growth (**Figure 4A**). Thus, BR and CO might be direct effectors of the HO1-regulated B tolerance.

It was reported that defects in *HY1* or *HY2* would cause a drop in chromophore production and reduced activity of all phytochrome species (Terry, 1997; Terry et al., 2002). To explore the possible interaction between light and the boron stress response, the *shb1* mutants were grown in the dark. However, their roots were still hypersensitive to excessive B similar to those grown in the light (**Figures 4B,C**). We then tested the high B response of phytochrome gene-related mutants (*phyAphyB*). In our hands, there was no obvious difference in primary root growth between the *phyAphyB* double mutant and the wild-type (**Figure 4D**). Our observations preliminarily suggest that *SHB1*, rather than phytochromes A and B, mediates Arabidopsis B tolerance. A similar result was obtained with the *hy5* mutant, which is defective in an important factor in the light signaling pathway (**Figure 4D**).

### HO1 Enzymatic By-Products Reduce B Accumulation in Roots

**FIGURE 5:**
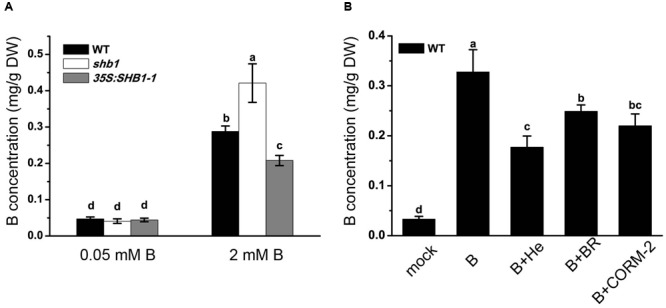
**Boron content analysis in roots of wild-type and *shb1* mutants.** Boron concentrations in roots **(A)** of wild-type, *shb1*, and *35S:SHB1-1* seedlings grown on medium containing 0.05 and 2 mM B for 21 days. **(B)** B concentrations in roots of wild-type seedlings grown on medium containing 0.05 and 2 mM B supplemented with 20 μM hematin (He), 50 μM CORM-2, 10 μM BR, and 50 μM Fe-EDTA for 21 days. All the data are shown as means ± SD (*n* = 3). Bars with different letters are significantly different at *P* < 0.05 according to Tukey’s multiple range test. DW, dry weight.

Boron content analysis proved that both *shb1* and *35S:SHB1-1* plants had similar levels of B in the roots as that of the wild-type when grown on normal medium (**Figure 5A**). However, when grown on medium containing 2 mM B, boric acid accumulation in *shb1* roots was 45.8% higher than that in wild-type roots, while that in the *35S:SHB1-1* was 27.8% lower (**Figure 5A**).

According to previous reports, the protective effect of HO1 in plant stress responses could be due to its catalytic products (Noriega et al., 2004; Balestrasse et al., 2005; Sa et al., 2007). We therefore tested the effects of hematin, CORM-2, and BR on reducing B levels in roots of wild-type seedlings grown on medium containing high levels of boron, and the results revealed that B content in wild-type roots was 0.33 mg/g dry weight (DW) when grown on medium supplemented with 2 mM B and decreased to 0.18, 0.25, and 0.22 mg/g DW with addition of hematin, CORM-2, and BR in the medium, respectively (**Figure 5B**), implying that the products of HO could significantly lower B accumulation in roots allowing plants to acquire tolerance of B toxicity.

### *SHB1/HY1* Decreases B Content in Roots via Induction of *BOR4* Expression

**FIGURE 6:**
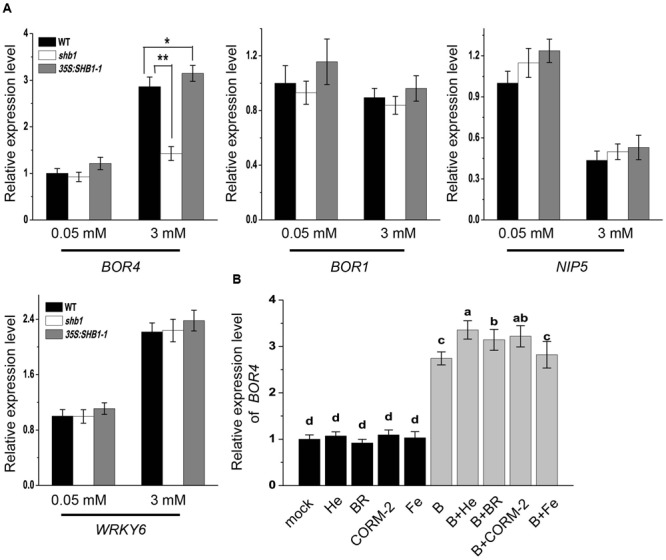
**Expression analysis of boron transport-related genes in roots. (A)** Quantitative RT-PCR analysis of *BOR4, BOR1, NIP5*, and *WRKY6* gene expression in wild-type, *shb1*, and *35S:SHB1-1* roots in response to boron toxicity. Five-day-old seedlings were transferred to medium containing 0.05 or 3 mM B and incubated for 2 days. Data were normalized to *ACTIN8* mRNA levels in the same samples. Data are shown as means ± SD (*n* = 3), and asterisks represent significant differences (^∗^*P* < 0.05, ^∗∗^*P* < 0.01, Student’s *t*-test) relative to wild-type. **(B)** Quantitative RT-PCR analysis of *BOR4* in roots under excessive B treatment with or without hematin (He), CORM-2, BR, and Fe supplementation. Data were normalized to *ACTIN8* mRNA levels in the same samples. Data are shown as means ± SD (*n* = 3). Bars with different letters indicate significant differences (*P* < 0.05) according to Tukey’s multiple range test.

Previous studies have revealed that plants regulate boron content through certain transporters and channels (Takano et al., 2008; Reid, 2014). We compared mRNA levels of some boron transporter and channel genes, including *BOR4, BOR1*, and *NIP5*, in wild-type, *shb1*, and *35S:SHB1-1* roots. *WRKY6* was also examined, which was reported to be induced by low B and plays a role under high B conditions (Kasajima et al., 2010). The expression patterns of *WRKY6* were not altered in either *SHB1*-related mutant (**Figure 6A**).

*NIP5* is known to be suppressed by high B levels, and there was no significant difference between wild-type and *SHB1*-related mutants under either normal or high B conditions (**Figure 6A**). *BOR1* mRNA accumulation was not regulated by high levels of B, and its mRNA levels were not affected by *SHB1* (**Figure 6A**). In contrast, *BOR4* could be up-regulated by high B treatment in the wild-type and *35S:SHB1-1* plants, but failed to be induced in *shb1* roots (**Figure 6A**).

In addition, application of hematin, CORM-2, and BR, but not Fe, in the medium further promoted *BOR4* expression in the wild-type under high B conditions (**Figure 6B**). These data suggested that *BOR4* might be downstream of *SHB1/HY1*, which should be necessary for high B-induced *BOR4* expression.

**FIGURE 7:**
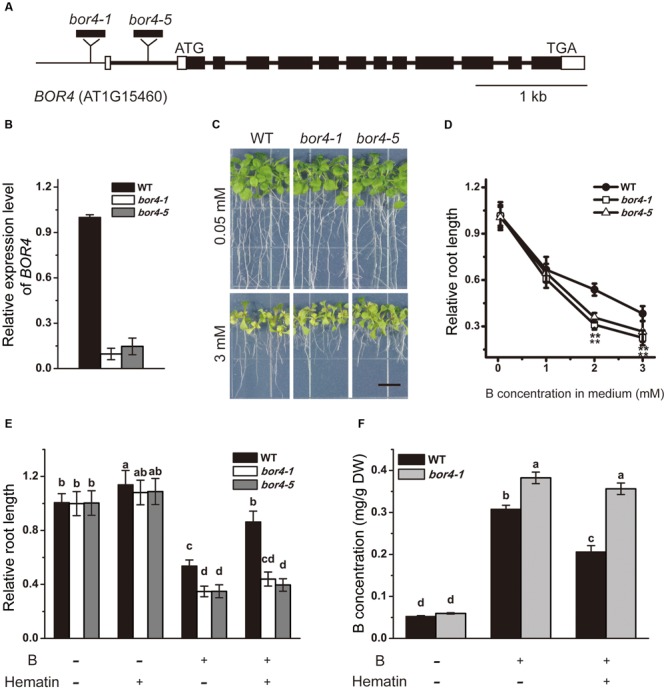
**Phenotype analysis of *bor4* mutants. (A)** Genomic structures of *BOR4*. Black and white boxes indicate coding regions and untranslated regions of *BOR4*, respectively. The sites for T-DNA insertion lines are shown. **(B)** Real-time PCR analysis of *BOR4* gene expression in wild-type, *bor4-1*, and *bor4-5* seedlings grown on normal medium. Total RNA was extracted from the whole seedlings of 10-day-old seedlings. Data were normalized to *ACTIN8* mRNA levels in the same samples. Data are shown as means ± SD (*n* = 3). **(C)** Phenotype of *bor4-1* and *bor4-5* mutant seedlings under excessive B treatment. Bars = 1 cm. **(D)** The relative root length of *bor4-1* and *bor4-5* seedlings grown on medium containing 0.05, 1, 2, or 3 mM B for 21 days. Asterisks represent significant differences (^∗∗^*P* < 0.01, Student’s *t*-test) relative to wild-type. **(E)** The relative root length of wild-type, *bor4-1*, and *bor4-5* seedlings grown on medium containing 0.05 or 3 mM B with or without 20 μM hematin for 21 days. Bars with different letters are significantly different at *P* < 0.05 according to Tukey’s multiple range test. **(F)** B content measurement in roots of wild-type and *bor4-1* seedlings grown as described in **(E)**. All the data are shown as means ± SD (*n* = 3). Bars with different letters indicate significant differences (*P* < 0.05) according to Tukey’s multiple range test. DW, dry weight.

To further explore the linkage between SHB1/HY1 and BOR4, we obtained two independent T-DNA insertion mutants, *bor4-1* and *bor4-5*, from ABRC (**Figures 7A,B**). According to a previous report, although the *BOR4* gene is expressed mainly in the roots, the *BOR4*-defective mutants (*bor4-1, bor4-2*, and *bor4-3*) accumulated higher levels of boron in shoots, and the shoots exhibited a striking phenotype (Miwa et al., 2014). In our hands, under long-term excessive B treatment, root growth of the *bor4-1* and *bor4-5* mutants was more sensitive compared with that in the wild-type (**Figures 7C,D**), and the boron content in *bor4-1* roots was significantly higher than that in the wild-type (**Figure 7F**).

As the HO1 inducer hematin could rescue the excessive B-induced root inhibition of the wild-type (**Figures 3D, 7E**), B content analysis in root tissues showed that the B level in wild-type roots was 0.31 mg/g DW when grown on medium supplemented with 2 mM B, and dropped to 0.21 mg/g DW with addition of hematin (**Figures 5B, 7F**). In contrast, application of hematin had no effect on *bor4-1* and *bor4-5* root growth (**Figure 7E**) and failed to reduce B accumulation in *bor4-1* roots (**Figure 7F**). These data suggested that HO1 promotes the exclusion of B in root cells by up-regulating the expression of the B transporter BOR4.

## Discussion

Excessive boron can be toxic to plants. Identification of genes related to the response to high boron levels will help us to understand the physiological fundamentals of boron toxicity. Sakamoto et al. (2009, 2011) isolated several Arabidopsis mutants with root growth defects on medium containing 3 mM B, and indicated that DNA damage is one of the toxic effects caused by excessive B and that the *HEB1* and *HEB2* genes might function in this process to promote tolerance of high B. Here, we showed that HO1 also plays a role in plant tolerance of boron toxicity.

### *SHB1/HY1* Is a Necessary Modulator in Mediating Plant Tolerance of Excess B Stress

In plants, numerous clues have accumulated highlighting that HO1 functions in response to various abiotic stresses. In this study, we provided evidence to demonstrate that HO1 is necessary in the regulation of boron exclusion and tolerance of excessive boron toxicity in plants.

The *shb1* mutant was identified for its hypersensitivity to excessive boron in root growth (**Figures 1A,B**). *SHB1* turned out to be the previously reported *HY1* gene, which encodes HO1 (**Figures 2A–C** and Supplementary Figures S2A–D).

There are four homologous *HO1* genes in the Arabidopsis genome (Gisk et al., 2010), and only *HO1* shows the highest transcriptional level in roots (Zimmermann et al., 2004; Xie et al., 2011). Single mutants of *HO2, 3*, or *4* did not show any hypersensitivity to high levels of B (Supplementary Figure S4), so it is most likely that HO1, rather than the other HOs, is a major player in plant tolerance to excess B stress in roots. We showed that promoting the expression of *SHB1/HY1* either by application of hematin or by constitutive over-expression of the *SHB1/HY1* gene resulted in greater resistance to excessive B treatment (**Figures 3D,E,G**).

It has been suggested that HO1 expression or/and activity could be induced by certain stress conditions, and its protective effect during plant stress responses is due to the products of its catalytic actions (Noriega et al., 2004; Balestrasse et al., 2005; Sa et al., 2007). Our results also supported this hypothesis that CO and BR can rescue the high-boron hypersensitivity of *shb1* (**Figure 4A**), while the CO scavenger hemoglobin eliminated the CORM-2 effect (**Figure 4A**).

We further explored the possibility of light being involved in the boron stress response. Even though we cannot completely rule out the involvement of light signaling in excess B tolerance in plants, the insensitive phenotypes of *phyAphyB* and *hy5* mutants under high boron stress (**Figures 4A–D**) suggested that decreased levels of the HO catalytic products CO and BR is the major cause of the compromised B stress tolerance in *shb1* roots.

### SHB1/HY1 Maintains B Content in Roots by Modulating the Expression of the Boron Efflux Transporter *AtBOR4*

It has been reported that excessive B can inhibit root growth (Sakamoto et al., 2009, 2011), and we found that boric acid content in *shb1* roots was higher than that in the wild-type under excessive B conditions (**Figure 5A**), implying that the short root phenotype might be due to the abnormally high accumulation of B in roots.

In response to B stress, many plant species can reduce B accumulation via root B exclusion (Hayes and Reid, 2004; Hamurcu et al., 2016), and the B efflux transporter *AtBOR4* has been reported to be involved in excluding excessive B to protect plants. The *BOR4* gene is inducible by high levels of B, and transgenic plants over-expressing the *BOR4* gene show significantly higher resistance to B than wild-type plants (Miwa et al., 2007, 2014; Kajikawa et al., 2011). By contrast, T-DNA insertion mutants of *BOR4* show a slight growth reduction under toxic levels of B (Miwa et al., 2014). A study of two different tomato hybrids with different B tolerance showed that the B-tolerant hybrid had significantly higher expression of *SlBOR4* in roots (Princi et al., 2016). These data indicate that the expression level of BOR4 might be correlated with plant B tolerance.

Our observation that the *BOR4* gene could no longer be induced by high B treatment in the *shb1* mutant, while increasing *SHB1* expression resulted in higher *BOR4* expression under high B conditions (**Figures 6A,B**), indicated that HO1 might be critical in regulating *BOR4* transcription and modulating B exclusion in roots. The lower level of *BOR4* expression could explain the high level of B accumulation in *shb1* roots.

Similar cases have been reported previously. For example, HO1 could maintain K^+^ homeostasis by up-regulating the transcription of the proton pump-encoding genes *AHA1, AHA2*, and *AHA3*, as well as *SOS1* (Bose et al., 2013). RNA sequencing analyses have also implied that *SHB1/HY1* is a necessary regulator in mediating gene expression changes under drought stress and most strongly activated or repressed genes in *hy1-100* were transport-related genes (Xie et al., 2016).

Carbon monoxide is a catalytic product of HO1. In plants, CO functions as a signaling molecule and has many functions in regulating root growth, stomatal opening and closing, and even reactive oxygen species balance. Li et al. (2013) showed that a solution saturated with CO gas could reverse leaf chlorosis in Arabidopsis and maize under iron deficiency conditions because the CO could promote the transcription of iron absorption-related genes such as *IRTI* in roots, thus increasing plant tolerance to iron deficiency stress (Li et al., 2013).

We also found that the HO1 catalytic products CO and BR could induce *BOR4* transcription, reduce B content in roots, and restore primary root growth under high B conditions in the wild-type and *shb1/hy1* mutants (**Figures 4A, 5B, 6B**). On the other hand, the CO scavenger hemoglobin inhibited the relief from high B toxicity (**Figure 4A**), implying that CO might play a major role in this process.

Our data, along with previous reports, led to the assumption that the catalytic products of HO1 are necessary regulators in plant stress responses and function by regulating the transcription of transport-related genes. However, pharmacological, genetics, and other analyses are still needed to further understand the relationship between CO, BR, and the regulation mechanism of B transporters.

### HO1 Might Be a Molecular Link in Regulating Different Abiotic Stress Responses

We noticed that mild B stress (2 mM), but not more severe B stress (4 mM), could induce *SHB1/HY1* mRNA accumulation after 24 h of treatment (**Figure 3A**). The promotion of *HY1* gene expression under mild B stress and the reduction in expression under severe B stress is similar to the pattern observed under salt stress treatment (Zilli et al., 2008; Xie et al., 2011) and might reflect the correlation between B toxicity and salinity stress.

Excessive accumulation of B in semi-arid and arid climates is frequently accompanied by salinity stress due to limited drainage. Therefore, crops are often subjected to both stresses simultaneously (Gupta et al., 1985; Grieve and Poss, 2000). Although, previous reports have indicated that salinity could intensify the effects of B toxicity in certain plant species (Grieve and Poss, 2000), the molecular mechanisms of the response to excessive B and salinity are largely unknown. Our results, together with previous reports regarding the involvement of *HY1* expression in salinity stress resistance (Zilli et al., 2008; Xie et al., 2011), suggest that a signal crosstalk exists in plants under both stresses, and heme oxygenase might be a hub involved in tolerance of both stresses.

## Author Contributions

Y-KH and FB conceived the project. QL and LW designed the experiments. J-ZW contributed the mutant screening and mapping. QL performed all experiments with the help of LW, PL, Y-LC, and JD. QL analyzed the data and wrote the article.

## Conflict of Interest Statement

The authors declare that the research was conducted in the absence of any commercial or financial relationships that could be construed as a potential conflict of interest.
