# Supplementary material for: SHB1/HY1 Alleviates Excess Boron Stress by Increasing BOR4 Expression Level and Maintaining Boron Homeostasis in Arabidopsis Roots
Source: Front Plant Sci. 2017 May 16;8:790. doi: 10.3389/fpls.2017.00790 (PMC5432644; doi:10.3389/fpls.2017.00790)
Supplement: Supplementary file 1 [file Presentation_1.PDF]

Supplementary Material

SHB1/HY1 Alleviates Excess Boron Stress by Increasing *BOR4* Expression Level and Maintaining Boron Homeostasis in Arabidopsis Roots

Qiang Lv[1]*, Lei Wang[1]*, Jin-Zheng Wang[1], Peng Li[1], Yu-Li Chen[1], Jing Du[1], Yi-Kun He[1] **and Fang Bao[1]**

[1] College of Life Sciences, Capital Normal University, Beijing, 100048, PR China

* Q.L. and L.W. contributed equally to the paper.

** To whom correspondence should be addressed.

Yi-Kun He: yhe@cnu.edu.cn

Fang Bao: 5838@cnu.edu.cn

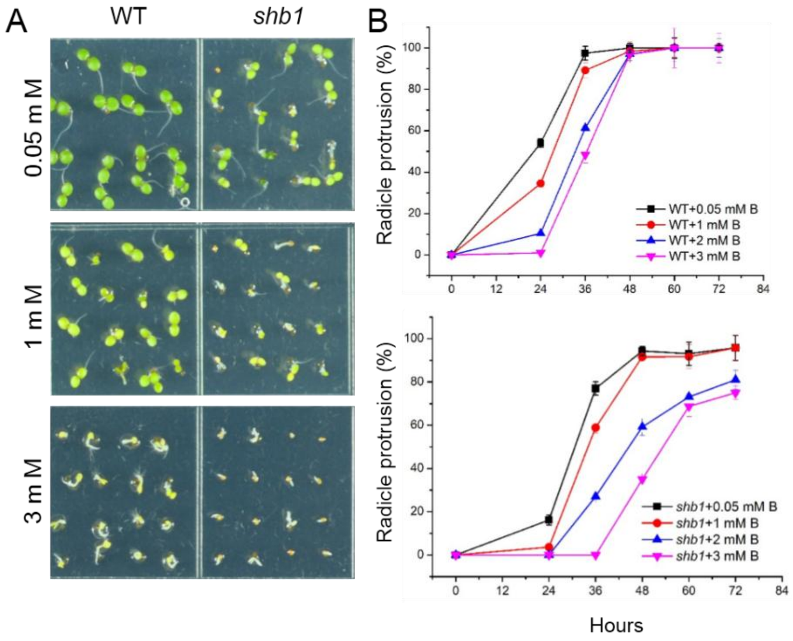

Figure S1. Seed germination under boron stress.

Phenotype **(A)** and germination time course **(B)** of wild-type (upper panel) and *hy1-100* (bottom panel) seeds in 0.05 (black) or in 1 (red), 2 (blue), 3 (purple) mM B.

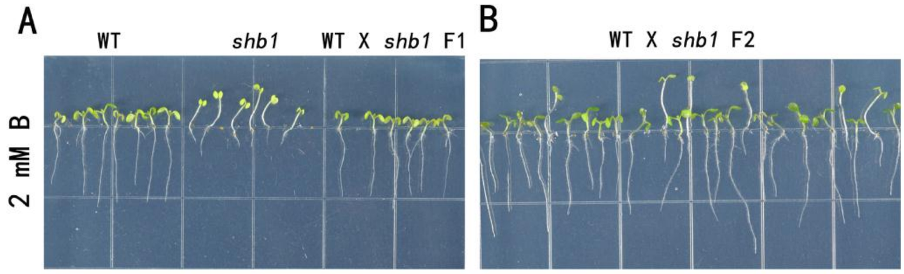

C

crosses	population	Number of plants		Segregation of ratio
		WT	*shb1*	WT/*shb1*
WT X *shb1*	F1	58	0	--
	F2	425	133	3.19
shb1 X WT	F1	73	0	--
	F2	515	177	2.91

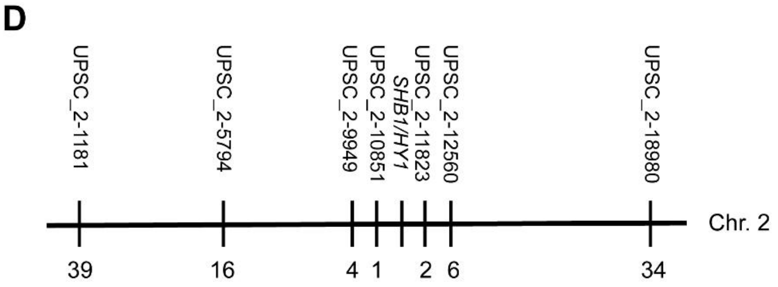

Figure S2. Genetic analysis and map-based cloning.
(A) Phenotype analyses of F1 hybrid seedlings of wild-type crossing with *shb1* under 2 mM B treatment. **(B)** Phenotypic segregation of the F2 generation under 2 mM B treatment. **(C)** Summary of boron resistent phenotype observed in the F1 and F2 generation of WT × *shb1* and *shb1* × WT crosses. These data were analyzed by $\chi 2$ test of goodness-of-fit of Mendelian ratios. **(D)** *SHB1* was mapped on chromosome 2 between marker UPSC_2-10851 and UPSC_2-11823.

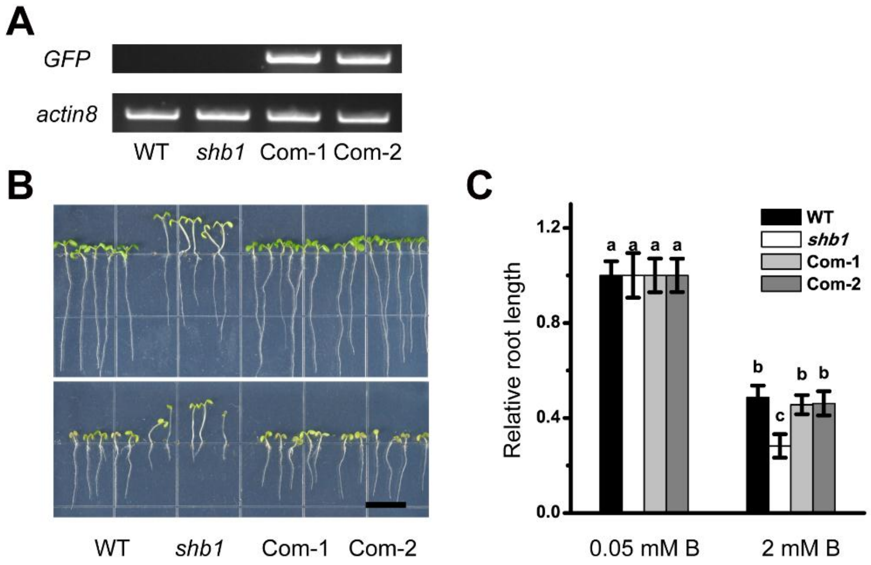

Figure S3. Complementation analysis of *shb1* Mutants.
(A) GFP expression levels in *SHB1$_{pro}$::SHB1:GFP* (*shb1*) plants analyzed by RT-PCR. Ten independent transgenic lines have been analyzed and line 1 (Com-1) and line 2 (Com-2) were shown. *AtACTIN8* was used as the reference gene. **(B)** Phenotypes of *SHB1$_{pro}$::SHB1:GFP* (*shb1*) plants. Plants were grown on medium containing 0.05 mM B (top) or 2 mM B (bottom) for 10 d. **(C)** Relative root length of wild-type, *shb1*, and transgenic lines. Values are means ± SD of three different experiments with at least three replicated measurements.

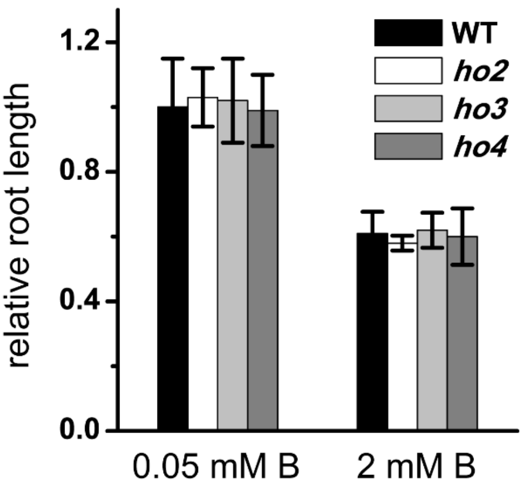

Figure S4. Excessive B-sensitivity analysis of HO mutants.

Excessive B-sensitivity analyses of single mutants of three HO encoding genes *HO2*, *HO3* and *HO4*. The data of relative root length of wild-type and the mutants are presented as means ± SD of three different experiments with at least three replicated measurements.

Supplemental Table 1. List of Primers Used in This Study

Primers for the mapping

Marker name	Type	Primer Sequence (5' to 3')	Product size	
			Col-0	Ler
UPSC_2-1181	SSLP	TCTCTGGTGTGATGTTGGAAA	97	114
		TTGGCACAACGAGATTTGAG		
UPSC_2-5794	SSLP	TCATGCGGAAGTGAGTGTTC	171	150
		TGCTTGAGTTTGGTTTTTGC		
UPSC_2-9949	SSLP	TTGGGTTTGAGTCACATTCG	211	171
		TACCTCCAACAAGCCACACA		
UPSC_2-10851	SSLP	CAATTGGGTACGCAAGATCA	130	147
		CACTCACTTTTTGCGTTTGC		
UPSC_2-11823	SSLP	CAAAAGCTTTCCTAATCCAGTG	109	119
		AAAAACTCTTGCGTCCTGGTT		
UPSC_2-12560	SSLP	CTCCAACACCACCTGCAA	186	150
		GAGATGGAGACCTGTTACGC		
UPSC_2-18980	SSLP	AAGCCTGGTTGTGGAACTTG	82	64
		CACCTTGCTTAACTGCCTTG		

Primers used for the detection of T-DNA insertion

Primer name	Sequences (5’→3’)
SALK_113008(ho2)-LP	GGCGAATAATACATAGACACG
SALK_113008(ho2)-RP	TGAGAAGAAACAAAGGAGCA
SALK_034321(ho3)-LP	GACTCAAAAGGTCAATCACA
SALK_034321(ho3)-RP	TTTTACAGCAAGATAGCCAC
SALK_044934(ho4)-LP	ATTGCAGTTTGCGTGTCTATT
SALK_044934(ho4)-RP	GTTCTTTCTCCGATTTCTCCT
SALK_135095(bor4-1)-LP	AATCCCGATCTACCATCCATG
SALK_135095(bor4-1)-RP	TTTGAAATTGCATATGCACCC
WiscDsLox233D10(bor4-5)-LP	AAAAACCAGACCAAACGTGG
WiscDsLox233D10(bor4-5)-RP	CTCTCCATTTGCTAACGTTGC
LBb1.3	ATTTTGCCGATTTCGGAAC

Primers for RT- PCR		Reference
q-Actin8-F	GCCAGATCTTCATCGTCGTG	Ohtsu *et al.*, 2004
q-Actin8-R	TCTCCAGCGAATCCAACCTT	
q-HY1-F	GAATCCCCAACTCTCAAG	Xie *et al*., 2011
q-HY1-R	TCATAGCCACAAACCTCA	
q-BOR4-F	GGAACTGTCTTTCCGGTCGAA	Miwa *et al.,* 2014
q-BOR4-R	CTTGGGATAAATCTGGTTGCCT	
q-BOR1-F	AATCTCGCAGCGGAAACG	Takano *et al.*, 2005
q-BOR1-R	TGGAGTCGAACTTGAACTTGTC	
q-NIP5-F	CACCGATTTTCCCTCTCCTGAT	Takano *et al.*, 2006
q-NIP5-R	GCATGCAGCGTTACCGATTA	
q-WRKY6-F	AGATGATCGAACGGACGTAAA	Kasajima *et al.*, 2010
q-WRKY6-R	CCATTTCGGAAGATTCTCCA	
RT-SHB1-F	GGTGGTTGCGGCTACTACTGC	
RT-SHB1-R	ACCTTTCTGCCAATCATTCGTC	
RT-EGFP-F	CAGTGCTTCAGCCGCTACCC	
RT-EGFP-R	AGTTCACCTTGATGCCGTTCTTCT	
RT-ACTIN 8-F	CTTCCACATGCTATCCTCCGTCTC	
RT-ACTIN 8-R	AGGACTTCTGGGCACCTGAATCTC	

REFERENCES

Kasajima,I., Idea,Y., Hiraib,M., and Fujiwara, T. (2010).WRKY6 is involved in the response to boron deficiency in Arabidopsis thaliana. *Physiol Plantarum*139, 80–92. doi: 10.1111/j.1399-3054.2010.01349.x

Miwa, K., Aibara,I., and Fujiwara, T. (2014). Arabidopsis thaliana BOR4 is upregulated under high boron conditions and confers tolerance to high boron. *Soil Sci Plant Nutr.*60, 349–355. doi: 10.1080/00380768.2013.866524

Ohkama-Ohtsu, N., Kasajima, I., Fujiwara, T., and Naito, S. (2004). Isolation and characterization of an Arabidopsis mutant that overaccumulates O-acetyl-L-Ser. *Plant Physiol.*136, 3209–3222.doi: 10.1104/pp.104.047068

Takano, J., Miwa, K., Yuan, L., von, Wirén. N., and Fujiwara, T. (2005).Endocytosis and degradation of BOR1, a boron transporter of Arabidopsis thaliana, regulated by boron availability.*Proc Natl Acad Sci USA.* 34, 12276-81.doi: 10.1073/pnas.0502060102

Takano, J., Wada, M., Ludewig, U., Schaaf, G., von, Wirén. N., and Fujiwara, T. (2006).The Arabidopsis major intrinsic protein NIP5;1 is essential for efficient boron uptake and plant development under boron limitation.*Plant Cell.*6, 1498-509.doi: 10.1105/tpc.106.041640

Xie, Y.J., Xu, S., Han, B., Wu, M.Z., Yuan, X.X., Han, Y. et al.(2011).Evidence of Arabidopsis salt acclimation induced byup-regulation of HY1 and the regulatory role of RbohD-derived reactive oxygen species synthesis.*Plant J.* 66, 280–292. doi: 10.1111/j.1365-313X.2011.04488.x
